# The IgG-to-Protein Ratio Influences the Relationship Between Brix Values and Immunoglobulin G Concentration in Bovine Colostrum

**DOI:** 10.3390/ani16192979

**Published:** 2026-09-22

**Authors:** Flávio G. Silva, Marta González-Cabrera, Lorenzo E. Hernández-Castellano, Rute Sobral, Ana Geraldo, Severiano R. Silva, Joaquim L. Cerqueira, Cristina Conceição

**Affiliations:** 1 MED-Mediterranean Institute for Agriculture, Environment and Development & CHANGE-Global Change and Sustainability Institute, University of Évora, Pólo da Mitra, Ap. 94, 7006-554 Évora, Portugal; fsilva@uevora.pt (F.G.S.); m53565@alunos.uevora.pt (R.S.); ageraldo@uevora.pt (A.G.); cristinaconceicao@uevora.pt (C.C.); 2 Veterinary and Animal Research Centre & Al4AnimalS, Department of Animal Science, Quinta de Prados, University of Trás-os-Montes e Alto Douro, 5000-801 Vila Real, Portugal; ssilva@utad.pt (S.R.S.); cerqueira@esa.ipvc.pt (J.L.C.); 3 Center for Research and Development in Agrifood Systems and Sustainability, Polytechnic Institute of Viana do Castelo, Agrarian School of Ponte de Lima, Rua D. Mendo Afonso, 147 Refóios do Lima, 4990-706 Ponte de Lima, Portugal; 4 IUSA-ONEHEALTH 4, Animal Production and Biotechnology Group, Institute of Animal Health and Food Safety, Universidad de Las Palmas de Gran Canaria, Campus Montaña Cardones, s/n, 35413 Arucas, Spain; marta.gonzalezcabrera@ulpgc.es

**Keywords:** soluble solids, IgG/CP ratio, passive immunity

## Abstract

This study investigated the relationship between Brix values and the nutritional and immunological composition of bovine colostrum. Colostrum samples (*n* = 82) were collected from Holstein-Friesian cows on four dairy farms. Dry matter (DM), solids non-fat (SNF), crude protein (CP), fat, IgG concentrations, and Brix values were determined. Correlation, regression, and receiver operating characteristic (ROC) analyses were used to evaluate associations among variables and identify a Brix cut-off for high-quality colostrum (IgG ≥ 50 g/L). Brix was positively associated with both CP (ρ = 0.90) and IgG concentration (ρ = 0.75), although the relationship between Brix and IgG was significantly influenced by the IgG/CP ratio. A robust regression model including SNF and fat provided the best prediction of Brix values (R^2^ = 0.98). ROC analysis identified a Brix cut-off of approximately 25%, which detected high-quality colostrum with 83% sensitivity and 76% specificity. Colostrum above this threshold had higher DM, SNF, CP, and IgG concentrations, but not fat concentration. These findings suggest that Brix values primarily reflect the soluble solids fraction of colostrum and that variation in the proportion of IgG within total protein may contribute to the variability in Brix–IgG relationships and threshold values reported among studies.

## Simple Summary

Newborn calves depend on colostrum, the first secretion produced by the mammary gland after calving, to obtain antibodies that protect them against infectious diseases. Farmers commonly use handheld Brix refractometers to estimate colostrum quality, as they are quick, inexpensive, and easy to use. However, different studies have reported different Brix values for identifying high-quality colostrum, creating uncertainty about their interpretation. This study investigated how Brix readings are related to the nutritional and immune components of bovine colostrum. Results showed that Brix values were strongly associated with the total amount of dissolved solids present in colostrum, particularly protein-rich components. The relationship between Brix values and antibody concentration depended on the proportion of antibodies within the total protein fraction, which may help explain the different Brix thresholds reported in previous studies. A Brix value of approximately 25% was the best indicator of colostrum containing high antibody concentrations in the studied population. Colostrum above this threshold also contained higher protein and dry matter concentrations, but not higher fat concentrations. These findings improve the understanding of how Brix refractometers work and support their use as practical tools for evaluating colostrum quality on dairy farms.

## 1. Introduction

Colostrum provides the calf with nutrients and bioactive components involved in energy metabolism and thermoregulation, as well as in the transfer of passive immunity (TPI) and gastrointestinal development [1]. As newborn calves face several physiological challenges at birth, including hypoglycemia and hypogammaglobulinemia, prompt colostrum intake is essential [2]. However, colostrum composition can be affected by several factors [3]; thus, regular monitoring of colostrum quality is important for an adequate colostrum management program [4]. Colostrum quality is primarily defined by IgG concentration [5] measured through either radial immunodiffusion (RID) [6,7] or enzyme-linked immunosorbent assay (ELISA) [8,9]. In addition, IgG concentration can be estimated by Brix refractometry [9,10], a quicker and more cost-effective on-farm tool than the laboratory gold standards [6,11]. The most common Brix cut-off for high-quality colostrum ranges between 21% and 23%, although other thresholds such as 19.3% [9] and 14% [11] have been suggested, based on the assumption that high-quality colostrum should have an IgG concentration ≥ 50 g/L [4]. Therefore, considerable uncertainty remains regarding the most appropriate Brix threshold for distinguishing between high- and low-quality colostrum. The type of assay used to measure IgG concentration (i.e., RID or ELISA), sample size, and sample characteristics are some of the possible sources of variation in the different proposed thresholds. In addition, this variation may arise because Brix refractometry measures total soluble solids rather than IgG alone. Consequently, the relationship between Brix and IgG concentration may be influenced by differences in colostrum composition, particularly by the proportion of IgG within the total protein fraction. Brix values are strongly associated with total protein concentration in colostrum, including whey proteins such as α-lactalbumin and β-lactoglobulin, whose concentrations, together with that of IgG, may vary among samples [2,7]. In fact, despite the suitability of Brix refractometry to estimate colostrum quality at the farm level [9,12], the suitability of Brix to evaluate colostrum quality has recently been questioned [13,14]. Given the widespread use of Brix refractometers on farms, a better understanding of the compositional factors influencing Brix measurements is of practical relevance. In particular, it remains unclear whether variation in the proportion of IgG within the total protein fraction contributes to the inconsistent Brix–IgG relationships and cut-off values reported among studies. It is hypothesized that Brix values are positively associated with colostrum dry matter (DM), solids non-fat (SNF), crude protein (CP), fat, and IgG concentrations, and that variation in the IgG/CP ratio influences the ability of Brix values to predict IgG concentration. Therefore, this study evaluated the relationship between Brix values and the nutritional and immunological composition of bovine colostrum, with special emphasis on the influence of the IgG-to-protein (IgG/CP) ratio on the Brix–IgG relationship.

## 2. Materials and Methods

This study was approved by the Ethics Committee for Animal Welfare (ORBEA) at Universidade Trás-os-Montes e Alto Douro (UTAD, Portugal) under reference 2664-e-DZ-2023.

### 2.1. Sample Collection

Eighty-two first-milking colostrum samples (50 mL) were collected from healthy primiparous (*n* = 39) and multiparous (*n* = 43) Holstein-Friesian cows from four dairy farms in the district of Évora, Portugal. Colostrum was milked within 12 h after parturition and processed according to each farm’s standard procedures. Samples were collected immediately before feeding the calf: 36 samples were raw colostrum, and 46 had been heat-treated (60 °C for 60 min) as part of the farm’s routine colostrum management. All samples were stored at −20 °C and later transported on ice to the Laboratory of Technology and Quality of Regional Products-MED at the University of Évora. Prior to analysis, samples were thawed overnight at 4 °C.

### 2.2. Brix Values

Brix values were measured with a digital refractometer (ORF-E, Kern, Albstadt, Germany). The optical lens was thoroughly cleaned with distilled water and dried between samples. Samples were analyzed in duplicate, and the mean Brix value was used for statistical analysis.

### 2.3. Chemical Composition and Immunoglobulin Concentration

Dry matter percentage was determined using the air oven loss-on-drying method in accordance with AOAC Official Method 925.23. Total nitrogen content was analyzed with a LECO Protein Analyzer (FP528, LECO Corporation, St. Joseph, MI, USA), using a nitrogen-to-protein conversion factor of 6.38 to calculate the CP in each sample, according to ISO 14891:2002 [15]. Colostrum fat percentage was measured using the Gerber method, following AOAC Official Method 2000.18-2004. SNF was calculated by subtracting fat from DM. Additionally, 5 mL of each sample was weighed to calculate its density (density = mass/volume). Colostrum IgG concentration was measured using a commercial ELISA kit (Bethyl Laboratories, Montgomery, TX, USA) according to the manufacturer’s instructions. The intra-assay and inter-assay coefficients of variation were 4.8 and 4.5%, respectively. The IgG/CP ratio was calculated as

$$\mathrm{IgG}/\mathrm{CP}\;\mathrm{ratio}\;(\%)=\frac{\mathrm{IgG}\times 100}{\mathrm{CP}\times \mathrm{Density}}$$

where density was used to express IgG and CP on the same volumetric basis, as described by Løkke et al. [16].

### 2.4. Statistical Analysis

Statistical analyses were performed with R (version 4.5.2) [17]. Spearman correlations and simple linear regression were used to assess the relationships between Brix values and colostrum components (DM, CP, SNF, fat, and IgG). Linear regression models were also fitted within five IgG/CP ratio classes (IgG/CP ≥ 50; IgG/CP ≥ 40 and <50; IgG/CP ≥ 30 and <40; IgG/CP ≥ 20 and <30; and IgG/CP < 20) to explore the influence of protein composition on the Brix–IgG relationship. Additionally, a linear regression model including Brix, the continuous IgG/CP ratio, and their interaction was fitted to formally assess whether the association between Brix and IgG concentration was influenced by the proportion of IgG within CP.

Multiple linear regression models were evaluated with Brix as the outcome variable and colostrum composition variables as predictors. Regression assumptions were assessed, including normality of residuals, homoscedasticity (Breusch–Pagan test), influential observations (Cook’s distance), and multicollinearity using the variance inflation factor (VIF ≥ 5). Because heteroscedasticity and influential observations were detected, robust multiple linear regression was used as the final modeling approach [18] using the “robustbase” package, version 0.99.7 [19]. Alternative models were compared based on model fit (R^2^), residual standard error (RSE), and multicollinearity diagnostics using the “performance” package, version 0.17.1 [20].

Receiver operating characteristic (ROC) analysis was performed using the “pROC” package, version 1.19.0.1 [21] to identify an optimal Brix cut-off for high-quality colostrum (IgG ≥ 50 g/L), using the Youden index to maximize sensitivity and specificity. The area under the curve (AUC) was calculated, and diagnostic performance was evaluated through confusion matrices, from which accuracy, sensitivity, and specificity were obtained. Diagnostic performance was also assessed for Brix thresholds previously reported in the literature. The stability of the ROC-derived threshold was evaluated by bootstrap resampling (2000 iterations), and the median optimal threshold and 95% bootstrap confidence interval were estimated.

Finally, one-way ANOVA was used to compare colostrum components between high- and low-quality groups defined by the selected Brix cut-off, with normality and homogeneity of variances assessed using the Shapiro–Wilk and Levene tests, respectively.

## 3. Results

### 3.1. Associations Between Brix and Colostrum Components

Three samples with very high viscosity were excluded from the analysis due to pipetting errors. Therefore, the results shown in this section were obtained from 79 colostrum samples. Colostrum component percentages varied considerably, especially those of fat and IgG (Table 1). The IgG/CP ratio exhibited substantial variation, with a ≈33% coefficient of variation.

The associations between Brix and all colostrum components determined are shown in a correlation matrix (Table 2). Brix was correlated with CP (ρ = 0.90), SNF (ρ = 0.83), DM (ρ = 0.79), and IgG (ρ = 0.75). Simple linear regression showed that Brix more accurately predicted CP than IgG concentration (Figure 1).

Regression models fitted within IgG/CP ratio classes suggested that the predictive capacity of Brix for IgG increased progressively with increasing IgG/CP ratios (Figure 2). This observation was confirmed by a significant interaction between Brix and the continuous IgG/CP ratio (β = 0.110 ± 0.012; *p* < 0.001), indicating that the slope of the Brix–IgG relationship increased as the proportion of IgG within total protein increased. Regression models fitted within IgG/CP ratio classes further illustrated this effect, with the predictive capacity of Brix for IgG generally increasing as the IgG/CP ratio increased (Figure 2). Likewise, CP showed the strongest correlation with IgG concentration (ρ = 0.76).

Three multiple robust regression models with Brix as the outcome variable were tested (Table 3), as DM, SNF, and CP showed substantial collinearity. A model including SNF and fat provided the best fit (R^2^ = 0.98; VIF < 1.3). Inclusion of CP alongside SNF resulted in severe multicollinearity (VIF > 50) and only a marginal improvement in model performance. Furthermore, IgG concentration was not a significant predictor of Brix values in any of the multiple regression models evaluated (*p* ≥ 0.228), despite its strong univariate correlation with Brix. Inclusion of heat treatment status (raw vs. heat-treated colostrum) as a covariate did not significantly affect Brix values in any of the multiple regression models evaluated (*p* ≥ 0.163) and did not alter the relationships between Brix and colostrum composition.

### 3.2. Brix Validation Tests

From the ROC analysis, a Brix cut-off value with the best combination of Se and Sp was identified (i.e., Brix = 25.1%). The AUC of 0.86 (95% CI: 0.77–0.94) indicated a very good ability to distinguish between low-quality (<50 g/L of IgG) and high-quality colostrum (≥50 g/L of IgG). Bootstrap resampling (2000 iterations) yielded a median optimal Brix threshold of 25.1% (95% bootstrap CI: 23.9–28.3%), indicating that the ROC-derived threshold remained centered around 25% across resampled datasets. Diagnostic test characteristics for the ROC-derived threshold (25.1%) and for previously proposed thresholds are shown in Table 4. Classification of colostrum using the 25.1% Brix cut-off resulted in higher (*p* < 0.001) concentrations of DM (28.1 ± 4.3 vs. 22.5 ± 3.3%), SNF (22.5 ± 2.6 vs. 18.4 ± 2.7%), CP (17.9 ± 2.4 vs. 12.8 ± 2.8%), and IgG (68.3 ± 24.5 vs. 38.0 ± 13.3 g/L), but not fat percentage (5.1 ± 3.4 vs. 4.2 ± 2.6%; *p* = 0.208).

## 4. Discussion

The reliability of Brix refractometry for assessing colostrum quality has been subject to recent debate [13,14]. This study demonstrated that Brix values can be used to estimate colostrum IgG concentration, although this relationship is influenced by the IgG/CP ratio. While various Brix cut-off values have been proposed to distinguish between poor- and high-quality colostrum, the present findings indicate that a Brix value of approximately 25% provided the best balance between sensitivity and specificity for identifying colostrum with an IgG concentration ≥ 50 g/L. Furthermore, this cut-off effectively detected colostrum with high DM, SNF, and CP percentages, but not fat.

The Brix refractometer was originally used to estimate sugar content as part of the quality analysis of agricultural products and alcoholic beverages [23]. This instrument was then used to assess colostrum quality in mares [24,25,26] and dairy cows [12,27]. Brix values are affected by all soluble molecules, including proteins and carbohydrates, and it was shown that the removal of low-molecular-weight solutes (<6 kDa; mainly carbohydrates) from the sample by dialysis decreased Brix values [28]. As described by Schalich et al. [28], removing the fat content from the sample does not affect Brix readings, which could be explained by the fact that lipids are insoluble and scatter visible light, consistent with the lack of correlation between Brix and fat observed in the present study. Nevertheless, fat contributed additional predictive value once CP or SNF was included in the model, as previously reported [16]. This may reflect the fact that fat varies independently of other colostrum components, as supported by the near-zero correlations observed between fat and both CP and SNF. Thus, fat may explain a portion of the residual variation in Brix values after accounting for CP or SNF, possibly through indirect composition or physical effects on refractometry measurements. Indeed, Brix refractometry appears to primarily estimate the soluble solids fraction of colostrum. Since CP is a major component of colostrum SNF, it is usually reported in the literature that Brix refractometry mostly estimates colostrum CP [11,16].

Since Brix refractometry reflects the soluble solids fraction rather than IgG specifically, variation in the composition of the protein fraction may influence the relationship between Brix and IgG concentration. Crude protein includes IgG as well as other proteins, including whey proteins such as α-lactalbumin and β-lactoglobulin, whose relative contribution may vary among colostrum samples [2,7]. Consequently, two samples with similar total protein concentrations, and therefore potentially similar Brix values, may contain different IgG concentrations if IgG represents a different proportion of total protein. This provides a biological interpretation for the observed influence of the IgG/CP ratio on the Brix–IgG relationship. In samples with a higher IgG/CP ratio, a greater proportion of the protein contributing to the soluble solids fraction is IgG, resulting in a stronger association between Brix and IgG concentration. Conversely, low IgG/CP ratios reflect a larger proportion of total protein represented by non-IgG proteins, reducing the capability of Brix refractometry to estimate IgG concentration specifically. Thus, the same Brix value does not necessarily correspond to the same IgG concentration across samples with different protein compositions. Importantly, the present study did not quantify individual non-IgG protein fractions, and therefore their specific contribution to the observed variation cannot be established directly. Nevertheless, the significant Brix × IgG/CP interaction provides direct evidence that the proportion of IgG within total protein is an important factor determining the strength of the Brix–IgG relationship.

The relationship between Brix and IgG concentration has shown considerable variability across studies, with correlation coefficients ranging from 0.64 [22] to 0.90 [11] and determination coefficients ranging from 0.43 to 0.80, respectively. The present results are in agreement with the above-mentioned range and are consistent with the moderate-to-high associations reported by Bielmann et al. [12] and Morrill et al. [29]. This variability may be partly attributed to differences in the method used to quantify IgG concentration. While most validation studies relied on RID, Hue et al. [11] suggested that ELISA may yield stronger associations with Brix. Although radial immunodiffusion (RID) is traditionally considered a reference method for the determination of IgG concentration in bovine colostrum, ELISA was selected in the present study because it provides a sensitive and specific quantitative measurement of IgG and was suitable for the analysis of the large number of samples included in this study. Nevertheless, this was not supported by the present results nor by those of Fahim and Imbabi [30], indicating that the influence of the quantification method on Brix–IgG associations remains unclear. Further studies directly comparing ELISA and RID in the context of Brix validation are therefore required.

The present results provide direct evidence that variation in the IgG/CP ratio influences the relationship between Brix and IgG concentration, as previously suggested [16]. The significant interaction between Brix and the IgG/CP ratio indicates that Brix values become more predictive of IgG concentration as IgG represents a larger proportion of total protein. This mechanism may partially explain the variability in Brix–IgG associations and threshold values reported among studies and populations. Indeed, higher reported IgG/CP ratios led to stronger associations [11], while those with a ratio similar to that in the present study yielded comparable results [16].

The optimal cut-off identified in the present study was higher than those previously reported, such as Brix 14% [11], Brix 18% [29], Brix 19.3% [9], Brix 21% [6], Brix 22% [12,31], and Brix 23% [22]. Lower thresholds increased sensitivity but substantially reduced specificity, leading to a greater risk of classifying low-quality colostrum as high quality. In contrast, the 25.1% threshold provided the best compromise between sensitivity and specificity and resulted in the highest overall diagnostic accuracy. A limitation of adopting a higher cut-off is that enough colostrum meeting this criterion may not always be available on farms. In such cases, supplementation with a colostrum replacer can be used to increase IgG supply [32]. The higher threshold identified in the present study may also reflect differences in protein composition among colostrum samples, as the relationship between Brix and IgG concentration was significantly influenced by the IgG/CP ratio. Nevertheless, the proposed cut-off effectively discriminated colostrum with higher CP, SNF, and IgG concentrations but failed to differentiate between colostrum with either low or high fat percentages. Consequently, applying reduced feeding volumes with high-Brix colostrum could lead to insufficient energy provision for the newborn calf [4,11]. Therefore, Brix refractometry should be regarded primarily as a tool for assessing immunological and protein-related colostrum quality rather than as a tool to assess its energetic value.

Recent evidence from a large multicenter study further supports the population-dependent nature of Brix thresholds. Meletis et al. [33] evaluated 591 bovine colostrum samples from 42 Holstein dairy herds using Bayesian finite mixture models and identified an optimal Brix cut-off of 19.62%, with 90% sensitivity and 85% specificity. This threshold was lower than the 25.1% identified in the present study, despite both studies aiming to discriminate colostrum with an IgG concentration of approximately 50 g/L. Differences in population characteristics, the analytical method used to quantify IgG, and statistical approaches for establishing diagnostic thresholds may contribute to this variability. These findings further support the need for external validation of Brix thresholds across independent populations before their widespread adoption.

Another factor that may contribute to variation in Brix measurements is sample storage. In a recent study, Westhoff and Mann [34] reported that bovine colostrum stored at −20 °C showed a 4.1–6.2% reduction in Brix values compared with fresh samples during 4 to 52 weeks of storage, whereas IgG concentrations decreased by approximately 8% only at selected time points after 32 weeks [34]. Although the storage conditions in the present study were standardized for all samples, the effect of freezing may have influenced the absolute Brix values. This is particularly relevant because Brix reflects soluble solids rather than IgG specifically, and storage-related changes in Brix may therefore affect the apparent relationship between Brix and IgG. Consequently, comparisons of Brix thresholds among studies should consider not only the population and IgG assay used but also sample storage conditions and duration.

From a practical perspective, these findings suggest that Brix refractometry can be used as a rapid on-farm tool to identify colostrum with higher protein and IgG concentrations, although this interpretation should consider the variability in colostrum protein composition. In particular, the proposed threshold of approximately 25% may be useful for identifying high-quality colostrum within populations with characteristics similar to those studied here, while lower thresholds may increase sensitivity at the expense of specificity. Therefore, Brix measurements can support on-farm decisions regarding colostrum selection and management but should be used as a screening tool rather than as a direct measurement of IgG concentration, and local or population-specific validation should be considered before adopting a specific cut-off.

Finally, a limitation of the present study is the relatively small sample size compared with several large-scale validation studies of Brix refractometry. Although bootstrap resampling supported the stability of a threshold close to 25% within the studied population, some variability was observed, indicating that the precise numerical value should be interpreted cautiously. Consequently, the proposed cut-off should be validated in larger and independent populations before broader recommendation. Moreover, this study included samples collected from multiple farms, cows of different parities, and both raw and heat-treated colostrum. While heat treatment did not significantly affect the relationships evaluated in the present study, potential clustering or management-related effects associated with farm and parity were not explicitly modeled and should therefore be considered when interpreting the results. In contrast, the strong associations observed between Brix values and colostrum composition, together with the small number of predictors retained in the final regression models, suggest that the main conclusions regarding the factors influencing Brix measurements are unlikely to be substantially affected by sample size.

## 5. Conclusions

This study demonstrates that the relationship between Brix and IgG concentration is influenced by the proportion of IgG within total colostrum protein. This finding provides a potential explanation for the variability in Brix–IgG associations and cut-off values reported in the literature. The strong association between Brix and solids non-fat concentration indicates that Brix primarily reflects the soluble solids fraction of colostrum, of which protein is a major component, rather than IgG concentration alone. A Brix value of approximately 25% effectively identified colostrum with higher IgG, crude protein, dry matter, and solids non-fat concentrations, but not fat concentration, within the studied population. Given the relatively small sample size and the uncertainty surrounding the estimated threshold, this value should be considered population-specific and should be externally validated in larger and independent populations before being recommended as a broadly applicable farm-level cut-off. Therefore, Brix refractometry should be regarded primarily as a tool for assessing the immunological and protein-related quality of colostrum rather than its energetic value.

## Figures and Tables

**Figure 1:**
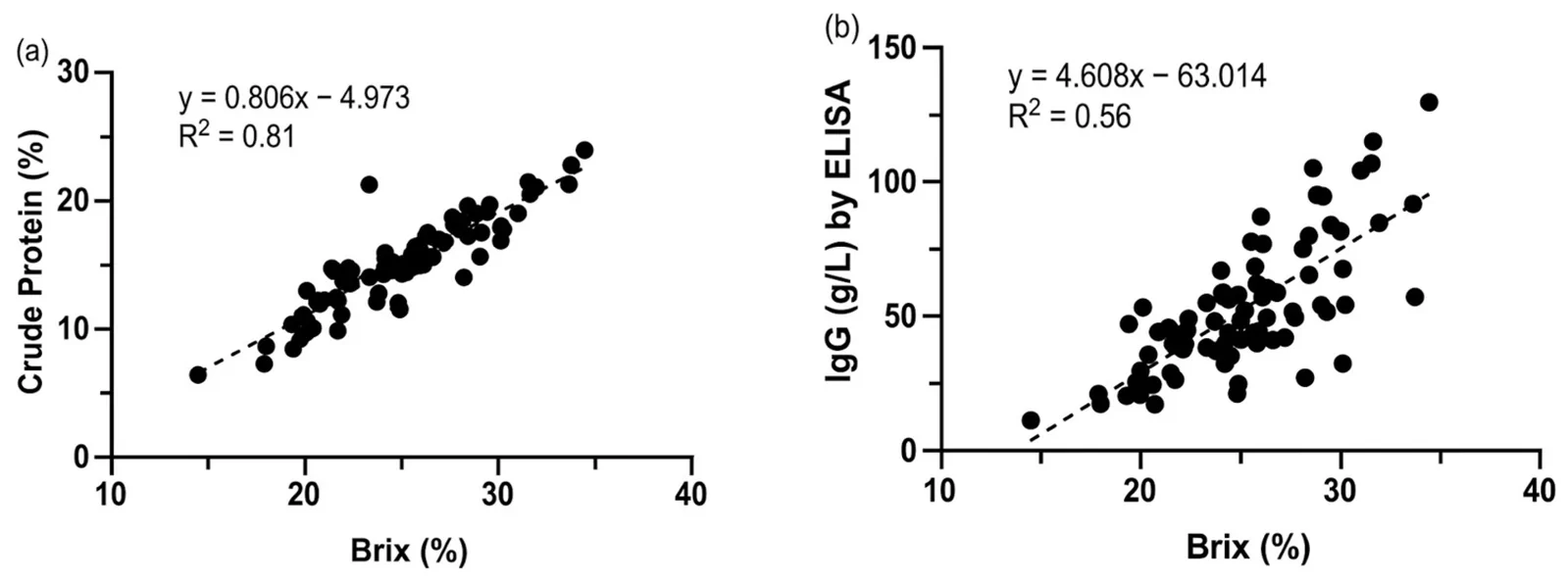
Linear regressions between Brix and crude protein (**a**) and between Brix and IgG (**b**).

**Figure 2:**
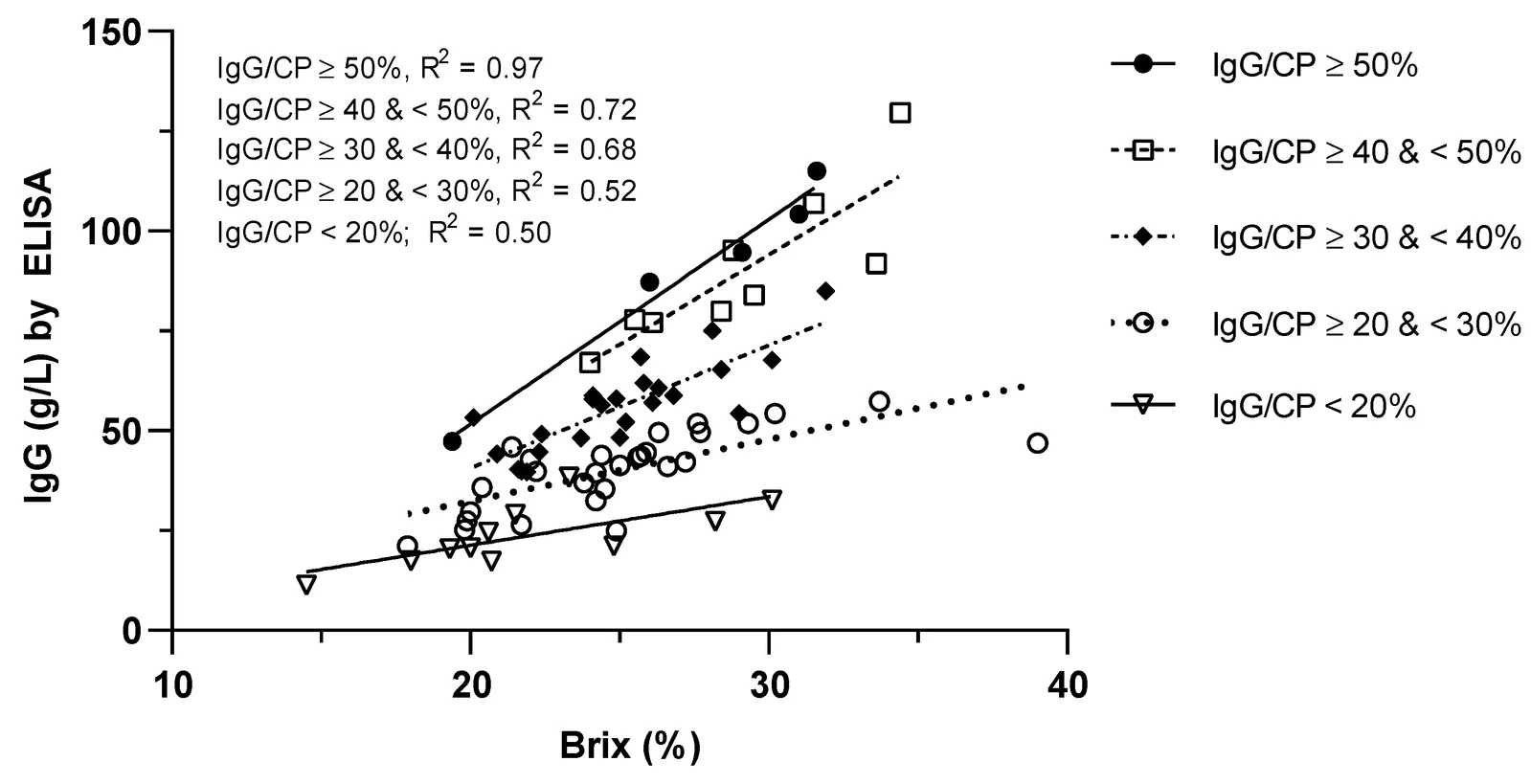
Linear regression between Brix and IgG in different classes according to the IgG/CP ratio (IgG/CP ≥ 50, *n* = 5; IgG/CP ≥ 40 and <50, *n* = 9; IgG/CP ≥ 30 and <40%, *n* = 24; IgG/CP ≥ 20 and <30, *n* = 28; and IgG/CP < 20%, *n* = 11). The increasing strength of the Brix–IgG relationship with increasing IgG/CP ratio was confirmed by a significant Brix × IgG/CP interaction in a continuous regression model (β = 0.110 ± 0.012; *p* < 0.001).

**Table 1:** Descriptive statistics for the nutritional and immunological parameters analyzed in bovine colostrum (*n* = 79).

Item	Mean	SD	Minimum	Median	Maximum
Brix (%)	25.3	4.3	14.5	25.0	39.0
DM (%)	25.2	4.9	13.2	25.1	35.7
SNF (%)	20.4	3.6	5.4	20.4	28.9
CP (%)	15.3	3.6	6.6	15.1	24.1
Fat (%)	4.6	3.0	0.9	3.8	12.0
IgG (g/L)	52.5	24.5	11.3	47.7	129.6
IgG/CP (%)	34.7	11.3	15.1	33.0	58.7

DM = dry matter; SNF = solids non-fat; CP = crude protein; IgG = immunoglobulin G.

**Table 2:** Spearman’s correlation matrix of the nutritional and immunological parameters analyzed in bovine colostrum.

	Brix	DM	SNF	CP	Fat
DM	0.79 ***				
SNF	0.83 ***	0.75 ***			
CP	0.90 ***	0.73 ***	0.90 ***		
Fat	0.19	0.60 ***	−0.08	−0.02	
IgG	0.75 ***	0.51 ***	0.67 ***	0.76 ***	−0.02

DM = dry matter; SNF = solids non-fat; CP = crude protein; IgG = immunoglobulin G. *** *p* < 0.001.

**Table 3:** Parameter estimates (β), standard errors (SE), and performance statistics of robust multiple linear regression models for Brix values.

Parameter	Model 1 (SNF + Fat)	Model 2 (CP + Fat)	Model 3 (SNF + CP + Fat)
Intercept	−0.919 (0.465)	7.781 (0.701) ***	0.829 (1.050)
SNF	1.210 (0.024) ***	-	0.938 (0.147) ***
CP	-	1.060 (0.040) ***	0.253 (0.135) ^†^
Fat	0.241 (0.030) ***	0.273 (0.040) ***	0.250 (0.026) ***
R^2^	0.980	0.920	0.982
RSE	0.65	1.10	0.64
VIFmax	1.22	1.01	54.44

^†^ *p* = 0.065; *** *p* < 0.001. SNF = solids non-fat; CP = crude protein; RSE = residual standard error; VIF = variance inflation factor.

**Table 4:** Diagnostic test characteristics for the digital Brix refractometer compared with the ELISA assay assessment (IgG ≥ 50 g/L).

Cut-Off Brix Value (%)	Accuracy (95% CI) ^1^	Se (95% CI)	Sp (95% CI)
25.1 ^a^	0.79 (0.69, 0.87)	0.83 (0.67–0.92)	0.76 (0.62–0.86)
23.0 ^b^	0.72 (0.61, 0.81)	0.97 (0.85–0.99)	0.52 (0.38–0.66)
22.0 ^c^	0.65 (0.54, 0.76)	0.97 (0.85–0.99)	0.41 (0.28–0.56)
21.0 ^d^	0.58 (0.47, 0.69)	0.97 (0.85–0.99)	0.28 (0.17–0.43)
19.3 ^e^	0.47 (0.36, 0.58)	1.00 (0.90–1.00)	0.07 (0.02–0.18)

Se = sensitivity; Sp = specificity; ^a^ best threshold obtained using the Youden index to maximize Se and Sp; ^b^ [22]; ^c^ [12]; ^d^ [6]; ^e^ [9]. ^1^ Se and Sp confidence intervals were calculated using Wilson’s method.

## Data Availability

The raw data supporting the conclusions of this article will be made available by the authors on request.

## Abbreviations

The following abbreviations are used in this manuscript:

AUC	Area under the curve
CP	Crude protein
DM	Dry matter
ELISA	Enzyme-linked immunosorbent assay
IgG	Immunoglobulin G
IgG/CP	Immunoglobulin G-to-crude protein ratio
RID	Radial immunodiffusion
ROC	Receiver operating characteristic
RSE	Residual standard error
SNF	Solids non-fat
TPI	Transfer of passive immunity
VIF	Variance inflation factor
